# Quick needle insertion at pharyngeal acupoints for poststroke dysphagia: A case report

**DOI:** 10.1097/MD.0000000000009299

**Published:** 2017-12-15

**Authors:** Xiaoning Li, Lei Wu, Fan Guo, Xuesong Liang, Hao Fu, Nuo Li

**Affiliations:** a Acupuncture and Moxibustion Four Ward, Second Affiliated Hospital of Heilongjiang University of Traditional Chinese Medicine; b Heilongjiang University of Traditional Chinese Medicine, Harbin, China.

**Keywords:** acupuncture, dysphagia, pharyngeal acupoints, stroke, swallowing

## Abstract

**Rationale:**

Dysphagia following stroke is a major complaint among patients, and effective treatment of post-stroke dysphagia can be difficult. We present a case report describing a new treatment for dysphagia, namely, quick needle insertion at pharyngeal acupoints.

**Patient concerns:**

A 70-year-old man developed pharyngeal dysphagia after a stroke. Three months after the patient experienced a sudden stroke leading to liquid dysphagia, acupuncture, one of the most important therapies in Traditional Chinese Medicine, was used to treat the patient.

**Diagnoses:**

A diagnosis of cerebral infarction and bulbar paralysis was made.

**Interventions:**

Quick needle insertion was performed at five pharyngeal acupoints, once a day, 6 times a week for 6 weeks.

**Outcomes:**

The patient subsequently showed significant improvement in the pharyngeal phase of swallowing. His performance in the drinking water test reduced to level 1 from level 4. The functional oral intake scale score changed from level 2 to level 7. In the video fluoroscopic swallowing study, no spillage occurred, but aspiration was present. The residue of the contrast agent was reduced.

**Lessons:**

Quick needle insertion at pharyngeal acupoints can be an efficient way to treat post-stroke dysphagia.

## 1 Introduction

Dysphagia is a major complaint following strokes. The clinical incidence rate of poststroke dysphagia is 37% to 78%. In turn, dysphagia causes malnutrition and/or dehydration in up to 25% of patients, and aspiration pneumonia in up to 20% of patients.^[1]^ A review in 2004 of 3200 ischemic stroke patients from South Carolina found that insurance costs for 1 year of treatment for patients with the same age, comorbidities, and race scores were $4510 less for those without dysphagia than for those with dysphagia.^[2]^ Thus, effective treatment of poststroke dysphagia is a challenge for rehabilitation physicians.

The current treatment options for poststroke dysphagia include swallowing training,^[3,4]^ repeated transcranial magnetic stimulation,^[5]^ transcranial electrical stimulation,^[6]^ and optimization of eating protocols.^[7]^ Acupuncture treatment of poststroke dysphagia has also achieved satisfactory results.^[8]^ In this case report, we describe a new treatment for dysphagia, namely, quick needle insertion at pharyngeal acupoints.

## 2 Case report

A 70-year old man was admitted to our hospital on March 20, 2017 with the chief complaints of coughing while drinking, dysphagia, and dysarthria, which had persisted for 1 month. The patient was suffering from bucking, swallowing difficulty, dizziness, and nausea. He had been vomiting brown food content at home since March 8 without obvious inducement. He was sent to The Second Affiliated Hospital of Harbin Medical University on March 9. Head magnetic resonance imaging showed an infraction in the left medulla oblongata on March 10. Magnetic resonance angiography revealed stenosis in the left vertebral artery, which was diagnosed as a “cerebral infarction.” After treatment for 12 days, the patient's clinical symptoms improved and he was discharged from the hospital. He came to our hospital for further treatment on March 20. Physical examination showed that the patient was conscious and had dysarthria, Horner sign (+) on the left side of the body, normal bilateral limb myodynamia, overactive tendon reflex on the right side, Babinski sign (+) on the right lower limb, and meningeal irritation sign (−). Consequently, a diagnosis of cerebral infarction and bulbar paralysis was made.

### 2.1 Assessment of swallowing ability

The drinking water test,^[9]^ functional oral intake scale (FOIS),^[10]^ and video fluoroscopic swallowing study (VFSS) were used to assess the patient's swallowing ability pre- and posttreatment.

#### 2.1.1 The drinking water test

The drinking water test is one of the most widely used methods for early screening of poststroke dysphagia in Chinese hospitals because it is a very convenient way for assessing the swallowing ability of a stroke patient. In this study, the patient was asked to drink 30 mL of warm water (37 °C), and the time taken for drinking and any instances of coughing are recorded. Swallowing ability is classified into 5 levels as follows: level 1 when the water can be swallowed smoothly in only 1 gulp within 5 seconds; level 2 when the water can be swallowed in 1 gulp, but takes more than 5 seconds, or in more than 2 gulps with no cough; level 3 when the water can be swallowed in 1 gulp, but with coughing; level 4 when need more than 2 gulps are needed to swallow the water, and there is coughing; and level 5 when the patient coughs often and cannot finish drinking the water.

#### 2.1.2 FOIS

The FOIS is a useful ordinal scale that reflects functional oral intake ability in dysphagia patients. It has adequate reliability, validity, and sensitivity and is a useful tool for documenting clinical change. It may be appropriate as an independent measure of functional oral intake in prospective studies of stroke-related dysphagia. Pretreatment, the patient's FOIS score was at level 2.

#### 2.1.3 VFSS

The VFSS is used to determine the presence or absence of aspiration. It has traditionally been the gold standard to assess swallowing difficulty. A 100 mL 60% w/v barium sulfate suspension was mixed with 3 g xanthan gum thickener. For safety reasons, the patient was allowed to drink only 5 mL of this mixture via a cup. Fluoroscopy was then performed in the lateral and posterior anterior positions to observe the patient's oropharyngeal state of activity.

### 2.2 Treatment methods

**Figure 1:**
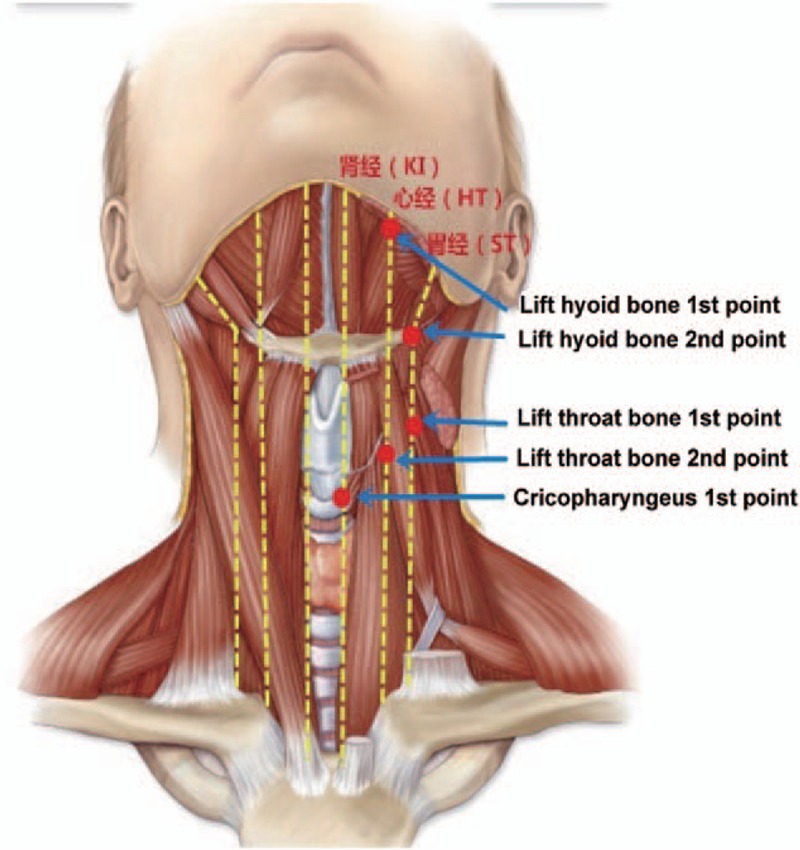
The acupuncture points.

The patient received some medication and rehabilitation training as treatment for the stroke. In addition, he received acupuncture treatment. The patient was treated once a day, 6 times per week for 6 weeks. Needles were inserted at the “Lift hyoid bone 1st point” (25 mm from the front midline, below the mandible), “Lift hyoid bone 2nd point” (behind the thyrohyal), “Lift throat bone 1st point” (under the thyroid cartilage, 13 mm from the prominentia laryngea, 25 mm from the front centerline), “Lift throat bone 2nd point” (50 mm from the intersection of the thyroid cartilage and the front midline), and “Cricopharyngeal 1st point” (5 mm from the intersection of the cricoid arch and the front midline) (Fig. 1).

ANDE brand disposable acupuncture needles (size 0.30 × 75 mm, Guiyang city, China) were used. After skin disinfection, the needles were inserted at a 10° angle. Needles were inserted approximately 12.5 mm into the skin at the 2 hyoid bone points and the cricopharyngeal point. Care was taken to avoid the carotid artery when needles were inserted at the second hyoid bone point.

### 2.3 Clinical outcomes

**Figure 2:**
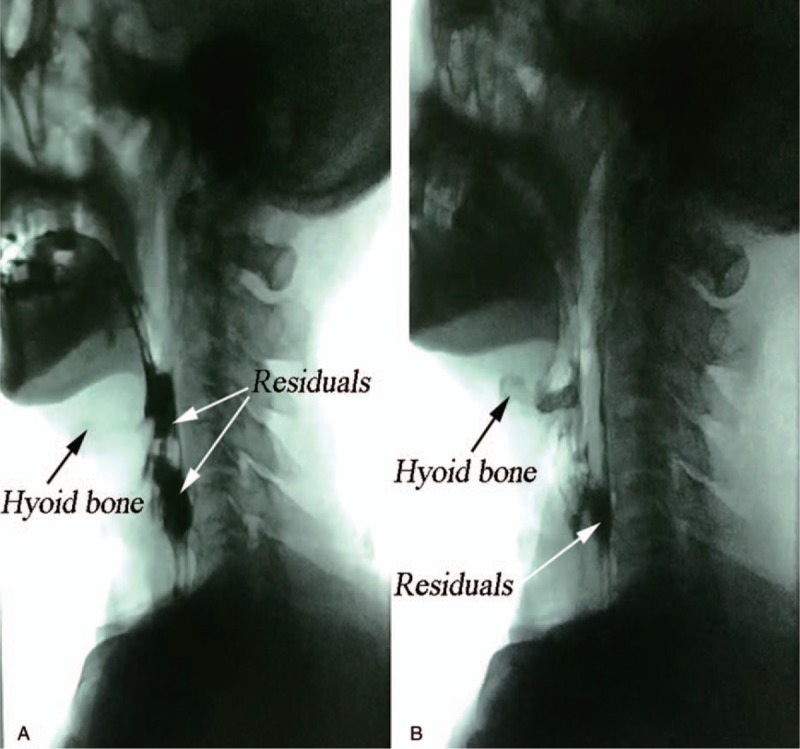
Residual bolus in the vallecula epiglottica and pyriform sinus. (A: pretreatment, B: posttreatment, black arrow represents hyoid bone and white arrow represents residuals).

The primary outcome was the improvement in the patient's ability to swallow, which was defined as improvement by 2 or more levels in the drinking water test and FOIS after treatment. After treatment, the patient's swallowing function improved significantly. His performance in the drinking water test reduced to level 1 (water can be swallowed smoothly in 1 gulp within 5 seconds) from level 4 (more than 2 gulps to swallow with coughing) (Table 1). The FOIS score changed from level 2 to level 7 (Table 2). In the VFSS, no spillage occurred, but aspiration was present. The residue of the contrast agent was reduced (Fig. 2 and Table 3).

**Table 1:**
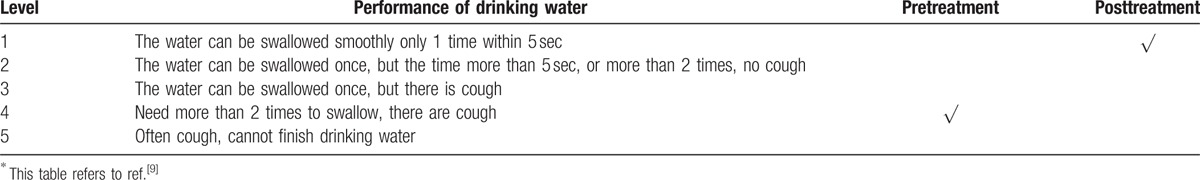
The drinking water test^∗^.

**Table 2:**
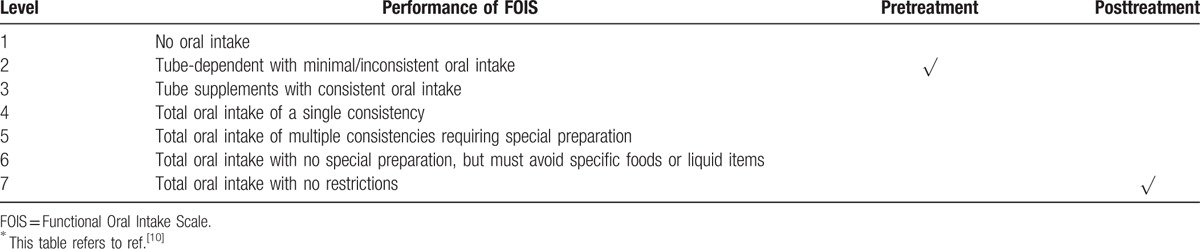
The FOIS^∗^.

**Table 3:**
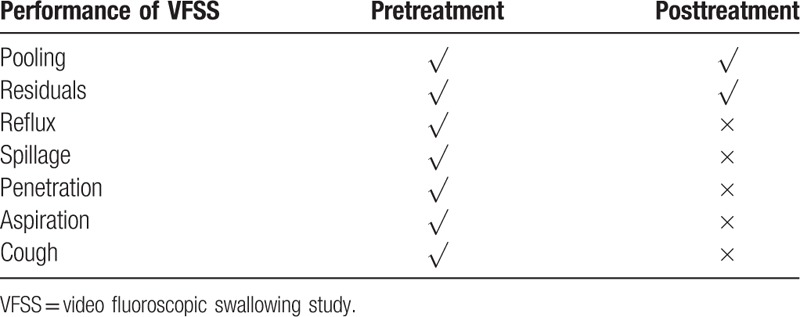
The VFSS result of the patient.

## 3 Discussion

Poststroke dysphagia is the consequence of damage at the pharyngeal cortex, which leads to incomplete laryngeal closure and failure of cricopharyngeal muscle relaxation.^[11]^ During swallowing, the hyoid bone moves up and down, providing the power to start the swallowing action. The movement of the hyoid bone occurs through vertical and anterior displacement. The vertical displacement of the hyolaryngeal structure contributes primarily to epiglottic and laryngeal closure, which protects the airway from penetration or aspiration, and anterior displacement contributes primarily to the opening of the upper esophageal sphincter, allowing passage of the bolus into the esophagus.^[12]^ The displacement of the hyoid bone occurs through muscle contractions. The geniohyoid muscle is responsible for anterior displacement, while the mylohyoid muscle is responsible for superior displacement.^[13]^ These muscles could be preferentially targeted for neuromuscular stimulation in the treatment of dysphasia.

Acupuncture can stimulate muscle contractions and has been reported to be effective in treating poststroke dysphagia.^[14]^ In this case report, a new treatment for dysphagia, namely, quick needle insertion at pharyngeal acupoints, was used. Stimulating these acupoints might make the geniohyoid and mylohyoid muscles contract, leading to the anterior and superior movement of the hyoid bone. We found that the patient's ability to drink water and his functional oral intake improved significantly after the treatment. In addition, aspiration disappeared, and the patient had a negligible amount of residual bolus in the vallecula epiglottica. We speculate that acupuncture treatment helps in the recovery of muscle function associated with swallowing. However, we did not use objective data, such as data on the displacement of the hyoid bone, to assess the efficacy of this treatment. This aspect will be explored in our future study.

In conclusion, quick needle insertion at pharyngeal acupoints is an efficient way to treat dysphasia after a stroke. It can reduce the residual of the barium sulfate and improve patients’ ability to drink water as well as their functional oral intake by stimulating the geniohyoid and mylohyoid muscles.

## References

[1] Rofes L Vilardell N Clavé P Post-stroke dysphagia: progress at last. Neurogastroenterol Motil 2013;25:278–82. 23480388 10.1111/nmo.12112

[2] Bonilha HS Simpson AN Ellis C The one-year attributable cost of post-stroke dysphagia. Dysphagia 2014;29:545–52. 24948438 10.1007/s00455-014-9543-8 PMC4179977

[3] Park JW Kim Y Oh JC Effortful swallowing training combined use electrical stimulation in post-stroke dysphagia: a randomized controlled study. Dysphagia 2012;27:521–7. 22447240 10.1007/s00455-012-9403-3

[4] Hägg M Tibbling L Effect of oral IQoro R and palatal plate training in post-stroke, four-quadrant facial dysfunction and dysphagia: a comparison study. Acta Otolaryngol 2015;135:962–8. 25947252 10.3109/00016489.2015.1042043

[5] Cheng IK Chan KM Wong CS Preliminary evidence of the effects of high-frequency repetitive transcranial magnetic stimulation (rTMS) on swallowing functions in post-stroke individuals with chronic dysphagia. Int J Lang Commun Disord 2015;50:389–96. 25588767 10.1111/1460-6984.12144

[6] Yang EJ Baek SR Shin J Effects of transcranial direct current stimulation (tDCS) on post-stroke dysphagia. Restor Neurol Neurosci 2012;30:303–11. 22572022 10.3233/RNN-2012-110213

[7] Vilardell N Rofes L Arreola V A comparative study between modified starch and xanthan gum thickeners in post-stroke oropharyngeal dysphagia. Dysphagia 2016;31:169–79. 26607158 10.1007/s00455-015-9672-8

[8] Mao LY Li LL Mao ZN Therapeutic effect of acupuncture combining standard swallowing training for post-stroke dysphagia: a prospective cohort study. Chin J Integr Med 2016;22:525–31. 27339160 10.1007/s11655-016-2457-6

[9] Zhao S-f He H Dou Z-l Cough reflex induced with citric acid in post-stroke dysphagia patients and healthy adults. Zhongguo Kangfu Lilun Yu Shijian 2015;21:567–71. (in Chinese).

[10] Crary MA Mann GD Groher ME Initial psychometric assessment of a functional oral intake scale for dysphagia in stroke patients. Arch Phys Med Rehabil 2005;86:1516–20. 16084801 10.1016/j.apmr.2004.11.049

[11] Kim SY Kim TU Hyun JK Differences in videofluoroscopic swallowing study (VFSS) findings according to the vascular territory involved in stroke. Dysphagia 2014;29:444–9. 24682308 10.1007/s00455-014-9525-x

[12] Kim Y McCullough GH Maximum hyoid displacement in normal swallowing. Dysphagia 2008;23:274–9. 17962998 10.1007/s00455-007-9135-y

[13] Pearson WG Jr Langmore SE Zumwalt AC Evaluating the structural properties of suprahyoid muscles and their potential for moving the hyoid. Dysphagia 2011;6:345–51. 10.1007/s00455-010-9315-z PMC3154991 21069388

[14] Cai H Ma B Gao X Tongue acupuncture in treatment of post-stroke dysphagia. Int J Clin Exp Med 2015;8:14090–4. 26550374 PMC4613059

